# Olivary subthreshold oscillations and burst activity revisited

**DOI:** 10.3389/fncir.2012.00091

**Published:** 2012-11-22

**Authors:** Paolo Bazzigaluppi, Jornt R. De Gruijl, Ruben S. van der Giessen, Sara Khosrovani, Chris I. De Zeeuw, Marcel T. G. de Jeu

**Affiliations:** ^1^Department of Neuroscience, Erasmus Medical CenterRotterdam, Netherlands; ^2^Netherlands Institute for Neuroscience, Royal Netherlands Academy of Arts and SciencesAmsterdam, Netherlands

**Keywords:** inferior olive, subthreshold oscillations, wavelets, climbing fiber, cerebellum, gap junctions

## Abstract

The inferior olive (IO) forms one of the major gateways for information that travels to the cerebellar cortex. Olivary neurons process sensory and motor signals that are subsequently relayed to Purkinje cells. The intrinsic subthreshold membrane potential oscillations of the olivary neurons are thought to be important for gating this flow of information. *In vitro* studies have revealed that the phase of the subthreshold oscillation determines the size of the olivary burst and may gate the information flow or encode the temporal state of the olivary network. Here, we investigated whether the same phenomenon occurred in murine olivary cells in an intact olivocerebellar system using the *in vivo* whole-cell recording technique. Our *in vivo* findings revealed that the number of wavelets within the olivary burst did not encode the timing of the spike relative to the phase of the oscillation but was related to the amplitude of the oscillation. Manipulating the oscillation amplitude by applying Harmaline confirmed the inverse relationship between the amplitude of oscillation and the number of wavelets within the olivary burst. Furthermore, we demonstrated that electrotonic coupling between olivary neurons affect this modulation of the olivary burst size. Based on these results, we suggest that the olivary burst size might reflect the “expectancy” of a spike to occur rather than the spike timing, and that this process requires the presence of gap junction coupling.

## Introduction

The inferior olive (IO) forms the sole source of climbing fiber inputs to Purkinje cells in the cerebellar cortex (Szentágothai and Rajkovits, 1959; Desclin, 1974). Climbing fibers excite Purkinje cells in the cerebellar cortex, resulting in a powerful, all-or-none depolarization called a complex spike (Eccles et al., 1966; Thach, 1967; Ito and Simpson, 1971). Climbing fibers may fire in bursts (Crill and Kennedy, 1967; Crill, 1970; Maruta et al., 2007; Mathy et al., 2009) and thereby they can modify the complex spike (Mathy et al., 2009). These climbing fiber bursts are generated at the axon hillock of olivary cells and they backpropagate to the soma where they give rise to small wavelets (Mathy et al., 2009).

IO neurons have two intrinsic properties that play an important role in their firing behavior: IO neurons generate subthreshold oscillations (Llinás and Yarom, 1986; Khosrovani et al., 2007) and they are coupled to one another via dendrodendritic gap junctions (Llinás et al., 1974; Sotelo et al., 1974; Khosrovani et al., 2007; Van Der Giessen et al., 2008). The subthreshold oscillations may serve as a timekeeping device, whereas the gap junctions (i.e., connexin 36) may be necessary to form functional ensembles of olivary cells (Llinás et al., 1974; Lang et al., 1996; De Zeeuw et al., 1998).

Recently, Mathy et al. (2009) have demonstrated that the burst activity of climbing fibers conveys information about the timing of the spike relative to the phase of the olivary subthreshold oscillations. The timing of the olivary activity was encoded by the number of spikes in the olivary axonal burst (i.e., climbing fiber burst). Furthermore, they showed that this climbing fiber burst (timing information) affected downstream Purkinje cells by altering the strength of the synaptic transmission between parallel fibers and Purkinje cells. Their results challenge current views concerning the role of climbing fibers in motor control, and they might reconcile the opposing theories established for motor timing and motor learning (Simpson et al., 1996; Mauk et al., 2000). However, an important part of their dataset was collected using whole-cell patch-clamp recordings from *ex vivo* slice preparations of the IO. Their *ex vivo* slice preparation had two major disadvantages; the olivary cells were isolated from their olivocerebellar module [which alters their electrophysiological behavior (Chorev et al., 2007; Khosrovani et al., 2007)] and the oscillations were artificially imposed to the neurons by injecting a fixed-amplitude sinusoidal current. Consequently, the finding that the climbing fiber signal encodes the temporal state of the olivary network could be due to the altered physiological condition of the IO neurons.

In the present study, we investigated whether this timing code was also generated in olivary cells present in an intact olivocerebellar system using the *in vivo* whole-cell recording technique. *In vivo* recordings were obtained under two different anaesthetic conditions to exclude drug-specific effects. Comparisons between spontaneous and somatosensory-evoked action potentials were made to elucidate origin-related differences. And we studied the consequences of genetically (connexin 36 knock-out mice: Cx36^−/−^) as well as pharmacologically manipulated (Harmaline) subthreshold oscillations on these olivary bursts.

## Materials and methods

### *In vivo* whole-cell recordings

C57 black 6 mice (C57BL/6 mice) were anesthetized by an intraperitoneal injection of a mixture of ketamine and xylazine (KX; 65 and 10 mg/kg; *n* = 40), or a mixture of medetomidine, midazolam, and fentanyl (MMF; 0.5 mg/kg, 5 mg/kg, and 0.05 mg/kg; *n* = 6). All Cx36^−/−^ mutants (*n* = 9) were anesthetized with KX. *In vivo* whole-cell recordings were performed as described by Khosrovani et al. (2007). In a subset of KX anesthetized animals (*n* = 4), peripheral stimulations were provided by electrical stimulation of the whisker pad to generate somatosensory evoked action potentials in the recorded olivary neuron. The stimulation protocol consisted of short bipolar stimulations (2 ms, 0.5 mA) that were randomly administered. In a second subset of KX anesthetized animals (*n* = 6), harmaline (50 mg/kg) was injected intraperitoneally after all baseline recordings were established. In each neuron, the following membrane properties were determined: input resistance, membrane capacitance (C_m_), resting membrane potential, and firing rate. The access resistance (R_a_), membrane resistance (R_m_), and C_m_ were calculated using the following formulas: R_a_ = *V*_Δ_/I_i_; R_m_ = (V_Δ_ − R_a_ I_ss_)/I_ss_ and C_m_ = τ(1/R_a_ + 1/R_m_). Tau (τ), instantaneous (I_i_), and steady state currents (I_ss_) were determined from current responses evoked by −10 mV steps. The resting membrane potential (V_m_) was determined from the readout of the baseline potential. All animal procedures were in accordance with the guidelines of the ethics committee of the Erasmus Medical Center.

### Data analysis

**Figure 1:**
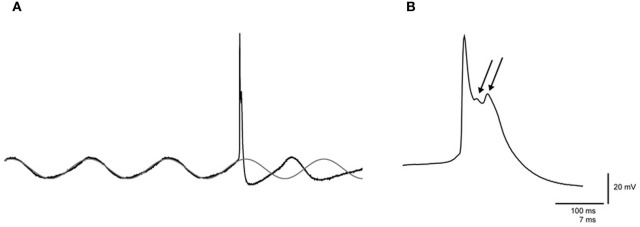
**Spontaneous sinusoidal subthreshold oscillations and wavelets of an olivary neuron. (A)** Left panel: trace of a spontaneous sinusoidal subthreshold oscillation (SSTO) from an olivary neuron *in vivo*. The gray trace indicates the sinusoidal fit of the SSTO prior to the occurrence of the action potential, followed by an extrapolation after the occurrence of the action potential. **(B)** Right panel: enlargement of the olivary action potential shown in the left panel. The arrows indicate the olivary wavelets on top of the afterdepolarization (ADP).

Data analyses were performed for neurons with resting membrane potentials lower than −45 mV, typical olivary spike waveforms (i.e., expressing spike afterdepolarizations with at least one wavelet), and spike amplitudes >60 mV (number of neurons: *n*_KX_ = 37, *n*_MMF_ = 8, *n*_Cx36^−/−^_ = 10). Subthreshold oscillations in the spontaneous membrane potential were quantified by measuring the frequency and amplitude of the oscillations. In neurons that expressed sinusoidal subthreshold oscillations (SSTOs), the phase spiking preference was determined by fitting the SSTO prior to the spontaneous action potential to a sine wave function. The fitted curve was extrapolated following the action potential, and spike occurrence was determined within phase bins of 45° (Figure 1). In neurons that expressed low-threshold calcium (Ca^2+^) oscillations (LTOs), the spike position was determined as either on top of the low-threshold Ca^2+^ spike or between them (Figure 8A). The number of wavelets on a typical olivary spike afterdepolarization (ADP) was counted for each spike.

### Statistical analysis

Statistical analysis of basic membrane properties was performed using Mann–Whitney *U*-test. The spiking preference in relation to the phase of the SSTO oscillation was examined using the Rayleigh test, and the shifts in the phase–frequency distributions under different conditions were compared using the Student's *t*-test. Modulations of the number of wavelets were tested by comparing the number of wavelets in each phase bin with the overall average number of wavelets using the one-sample Student's *t*-test. For bimodal analysis of the spiking preference in neurons expressing LTOs, the χ^2^ test was used. Modulations of the number of wavelets in these LTO neurons were analyzed statistically using the Student's *t*-test. Student's *t*-tests were also employed to test the significance of the correlation coefficients. For statistical comparisons of the linear regression lines, we used the analysis of covariance (ANCOVA) followed by a *post hoc* Tukey's HSD analysis (Matlab, The Mathworks, Natick, MA). All of the numerical values presented in the text represent the mean ± SEM.

## Results

### The generation of olivary wavelets *In vivo* does not depend on the phase of sinusoidal subthreshold oscillations

*In vivo* whole-cell patch-clamp recordings were obtained from neurons of the mouse IO. These recordings were collected under two different anaesthetic conditions induced by either KX or MMF to exclude anaesthesia-specific effects. Olivary neurons that were recorded under KX and MMF conditions revealed similar basic membrane properties (Table 1), and these properties were comparable to those obtained in previous *ex vivo* measurements (Llinás and Yarom, 1981; Long et al., 2002; De Zeeuw et al., 2003; Leznik and Llinas, 2005). However, the input resistance was slightly, but not significantly, higher under MMF conditions.

**Table 1:** **Membrane properties of *in vivo* olivary neurons under different anaesthetic and genetic conditions**.

**Membrane properties**	**KX anaesthesia (*n* = 37)**	**MMF anaesthesia (*n* =8)**	Cx36^−/−^ mutants^*^ (*n* = 10)	***p* value**
Rm (MΩ)	28.8 (22.3–38.2)	39.1 (31.7–41.8)	41.2 (39.8–49.7)	*p*_(KX−Cx36)_ < 0.05
Cm (pF)	207.5 (142.4–277.1)	203.5 (198.3–222.1)	130.9 (113.1–152.6)	*p*_(KX−Cx36)_ < 0.05
				*p*_(MMF−Cx36)_ < 0.05
Vm (mV)	−54.0 (−56.8 to −51.0)	−55.0 (−48.0 to −56.0)	−52.8 (−49.9 to −54.4)	ns
f (Hz)	0.44 (0.20–0.58)	0.24 (0.12–0.44)	0.47 (0.39–0.59)	ns

Statistical analyses of basic membrane properties were performed using the Mann–Whitney U-test.

* Cx36^−/−^ mutant mice were anaesthetized using the KX mixture.

Rm, membrane resistance; Cm, membrane capacitance; Vm, membrane potential; f, firing frequency. All numerical values indicate the median (25–75^th^ percentile).

**Figure 2:**
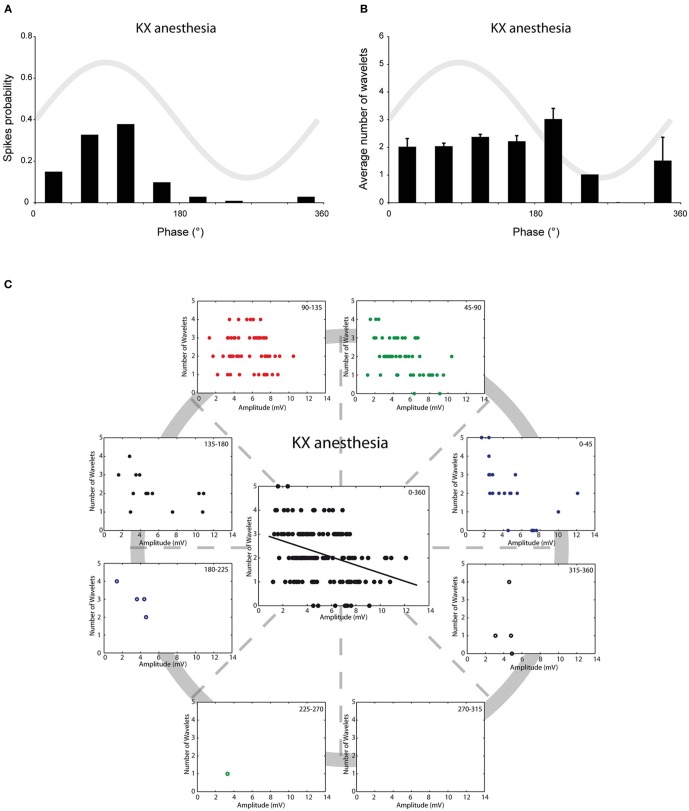
**Burst size of olivary wavelets and SSTOs under KX anaesthesia. (A)** The occurrence of spontaneous spikes (spike probability) in relation to the phase of the SSTO obtained from olivary neurons recorded under KX anaesthesia (*n* = 155). **(B)** Average number of wavelets on the ADP of spontaneous spikes in relation to the phase of the SSTO obtained from olivary neurons recorded under KX anaesthesia (*n* = 155). **(C)** Relationship between number of wavelets and amplitude of SSTO were plotted for each phase-bin. The center graph shows the correlation between the amplitude of the SSTOs and the number of wavelets of all data (all phase-bins together) measured under KX anaesthesia. Least squares linear regression lines and correlation coefficients were computed [*r*_KX_ = −0.36, *n* = 155, and *p* < 0.01 (*t*-test)].

**Figure 3:**
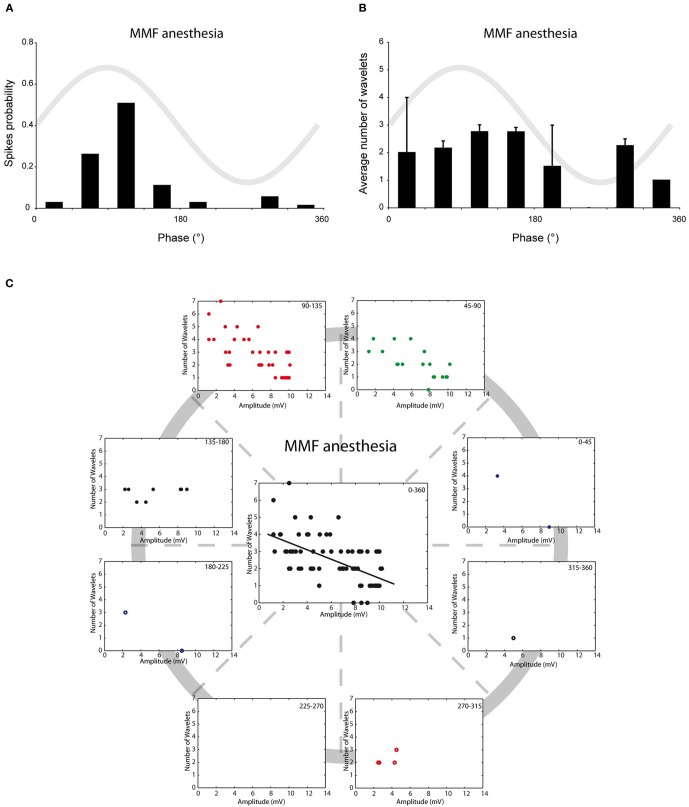
**Burst size of olivary wavelets and SSTOs under MMF anaesthesia. (A)** The occurrence of spontaneous spikes (spike probability) in relation to the phase of the SSTO obtained from olivary neurons recorded under MMF anaesthesia (*n* = 73). **(B)** Average number of wavelets on the ADP of spontaneous spikes in relation to the phase of the SSTO obtained from olivary neurons recorded under MMF anaesthesia (*n* = 73). **(C)** Relationship between number of wavelets and amplitude of SSTO were plotted for each phase-bin. The center graph shows the correlation between the amplitude of the SSTOs and the number of wavelets of all data (all phase-bins together) measured under MMF anaesthesia. Least squares linear regression lines and correlation coefficients were computed [*r*_MMF_ = −0.58, *n* = 73, and *p* < 0.01 (*t*-test)].

We have previously shown that *in vivo* olivary neurons can exhibit two different types of subthreshold oscillations: typical rhythmic 3–9 Hz SSTOs, or rhythmic 1–3 Hz LTOs (Khosrovani et al., 2007). Spontaneous action potentials in olivary cells that exhibited SSTOs depend on the peak phase of the oscillation under both anaesthetic conditions (both *r* = 0.71, both *p* < 0.05), but the phase-frequency distribution of spikes under MMF was slightly shifted compared to that under the KX condition (Figures 2A and 3A; KX: 98 ± 5°, MMF: 119 ± 7°; *p* < 0.05). To find out whether the phase of the subthreshold oscillations determines the number of wavelets in an olivary spike *in vivo*, we counted the number of wavelets that were superimposed on the spike ADP (Figure 1B; see arrows) and examined their dependency on the phase and amplitude of the subthreshold oscillation. On average, olivary spikes expressed 2.2 ± 0.1 wavelets (*n* = 155 spikes) under KX anaesthesia and 2.5 ± 0.2 wavelets (*n* = 73 spikes) under MMF anaesthesia. These values were not significantly different (*p* = 0.08) and were comparable to those measured *ex vivo* (Mathy et al., 2009). The timing of the spike in relation to the phase of the SSTO did not determine the number of olivary wavelets in either of the two conditions (Figures 2B and 3B; all *p* > 0.05). Inspection of the data of each neuron separately also did not reveal any correlation between the timing of the spike and the number of olivary wavelets. Thus, across the phase bins in which spikes occurred, there was no clear dependence of wavelet number on oscillation phase in our *in vivo* preparation. Alternatively, the amplitude of oscillation might be able to impose a modulatory effect on the number of wavelets. Therefore, we correlated the number of wavelets with the amplitude of the oscillation. This analysis was performed using data collected under both types of anaesthesia (Figures 2C and 3C). Significant correlations between the amplitude of the SSTO and the number of wavelets were detected for both anaesthetic conditions (Figures 2C and 3C, all phase-bins together, *r*_KX_ = −0.36, *r*_MMF_ = −0.58, both *p* < 0.01). The overall correlations revealed a negative relationship between the amplitude of the oscillation and the number of wavelets. Furthermore, the phase subset plots of Figures 2C and 3C show that there is no number of wavelet preferences between the different phase-bins.

### Spontaneous vs. somatosensory-evoked action potentials

**Figure 4:**
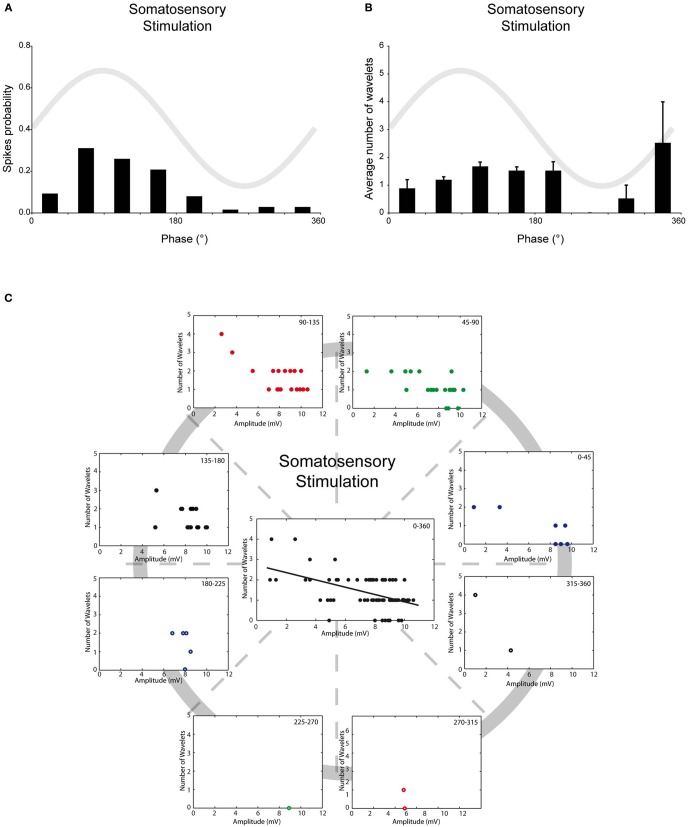
**Burst size of olivary wavelets and SSTOs of somatosensory-evoked spikes. (A)** The occurrence of stimulus evoked spikes (spike probability) in relation to the phase of the SSTO obtained from olivary neurons recorded under KX anaesthesia (*n* = 78). **(B)** Average number of wavelets on the ADP of stimulus evoked spikes in relation to the phase of the SSTO obtained from olivary neurons recorded under KX anaesthesia (*n* = 78). **(C)** Relationship between number of wavelets and amplitude of SSTO were plotted for each phase-bin. The center graph shows the correlation between the amplitude of the SSTOs and the number of wavelets of all data (all phase-bins together) measured on somatosensory evoked spikes. Least squares linear regression lines and correlation coefficients were computed [*r*_SSS_ = −0.49, *n* = 78, and *p* < 0.01 (*t*-test)].

Up to now, all the analyses were performed on spontaneous action potentials. Spike triggering inputs of olivary neurons can activate a subset of cellular responses that might alter the relationship between SSTO, spikes and wavelets. Therefore, we also investigated the relationship between SSTO and wavelets in somatosensory-evoked action potentials. Although strong stimuli were applied randomly to the mouse whisker pad, somatosensory-evoked action potentials in olivary cells that exhibited SSTOs were more easily generated at the peak of the oscillation, indicating also under this condition a clear spiking preference (Figure 4A, *r* = 0.57, *p* < 0.05, *n* = 78). The phase-frequency distribution of somatosensory-evoked spikes was slightly shifted compared to the spontaneous spikes under the KX condition (Figures 2A and 4A; spontaneous: 98 ± 5° and evoked: 119 ± 8°; *p* < 0.05). On average, the evoked olivary spikes expressed 1.4 ± 0.1 wavelets (*n* = 78 spikes), which is significantly smaller than the amount expressed on spontaneous spikes (2.2 ± 0.1 wavelets; *p* < 0.05). However, it is important to note that our somatosensory-evoked action potential group contain more recordings of cells with bigger SSTO amplitudes (see below). The timing of the evoked spike in relation to the phase of the SSTO did not determine the number of olivary wavelets (Figure 4B; all *p* > 0.05); indicating that the phase of the oscillation did not modulate the number of olivary wavelets on these spikes either. The amplitude of the oscillation and number of wavelets of these evoked spikes were significantly correlated to each other (Figure 4C, all phase-bins together, *r*_SSS_ = −0.49, *p* < 0.01), indicating also an inverse relationship between the amplitude of the oscillation and the number of wavelets on somatosensory-evoked spikes. Overall, we conclude that under *in vivo* conditions, the phase of the SSTO does not regulate the number of wavelets (i.e., output) on olivary spikes, but that the number of wavelets depends on the amplitude of the SSTO. This phenomenon can be observed on both spontaneous as well as somatosensory-evoked action potentials, despite their different origin and the activation of different cellular responses.

### Electrical synapses

**Figure 5:**
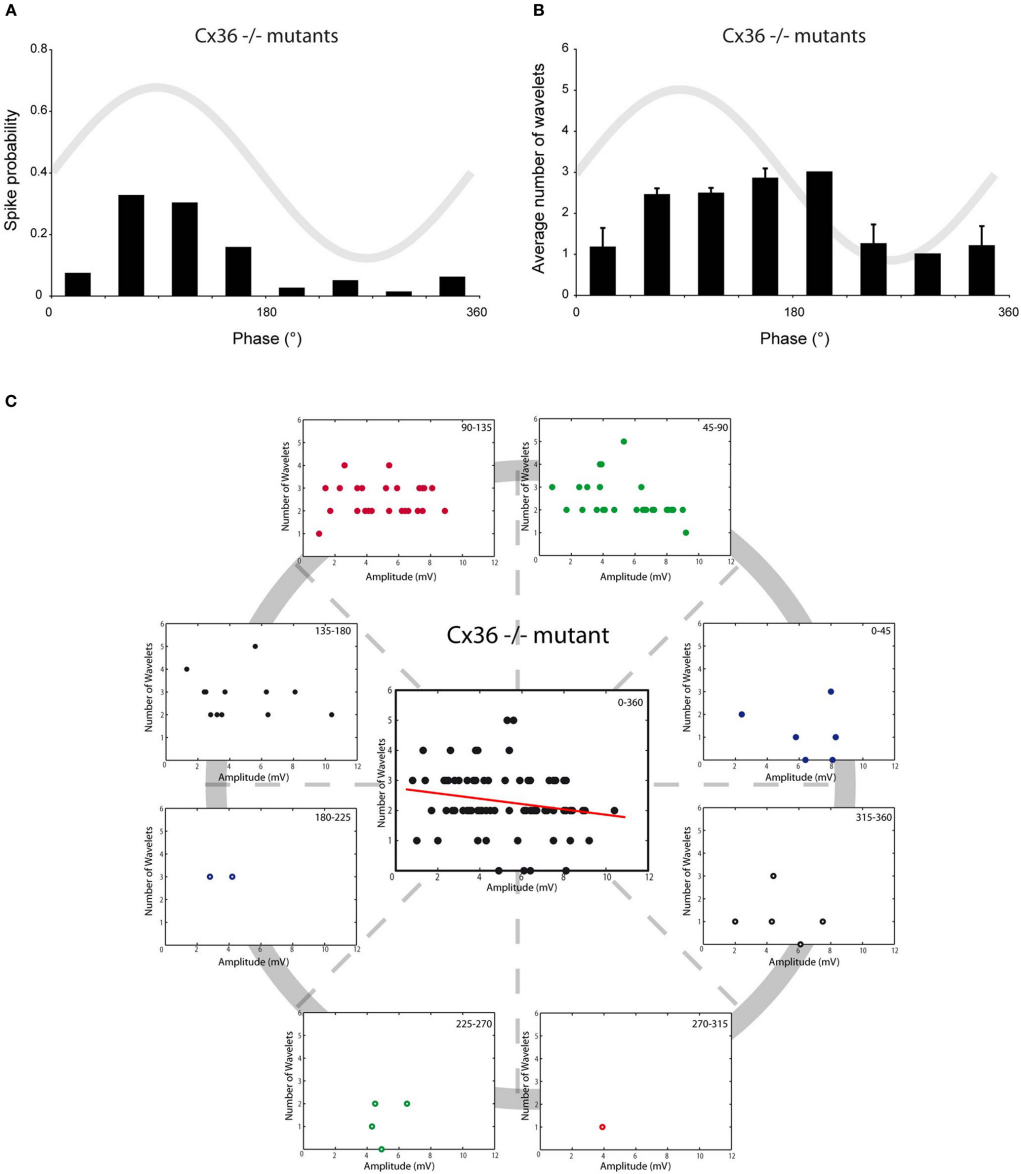
**Burst size of olivary wavelets and SSTOs in Cx36^−/−^ mutants. (A)** The occurrence of spontaneous spikes (spike probability) in relation to the phase of the SSTO obtained from olivary neurons recorded in Cx36^−/−^ mutants (*n* = 83). **(B)** Average number of wavelets on the ADP of spontaneous spikes in relation to the phase of the SSTO obtained from olivary neurons recorded in Cx36^−/−^ mutants (*n* = 83). **(C)** Relationship between number of wavelets and amplitude of SSTO were plotted for each phase-bin. The center graph shows the correlation between the amplitude of the SSTOs and the number of wavelets of all data (all phase-bins together) measured in Cx36^−/−^ mutant mice. Least squares linear regression lines and correlation coefficients were computed [*r*_CX36_ = −0.20, *n* = 83, and *p* > 0.05 (*t*-test)].

Olivary neurons are interconnected via gap junctions formed by connexin 36 (Cx36). The lack of Cx36 leads to an absence of electrotonic coupling, to a more voltage-dependent SSTO, to an increased excitability at hyperpolarizing states and to an altered interaction between SSTO and the generation of an action potential (Long et al., 2002; De Zeeuw et al., 2003; Van Der Giessen et al., 2008). Therefore, Cx36^−/−^ mutant mice provide a condition in which the relationship between the generation of action potentials and the phase of the oscillation is weakened, which might give the opportunity to unmask possible phase-related modulatory effects on olivary burst firing. *In vivo* whole-cell recordings from the SSTO neurons of Cx36^−/−^ mutants anesthetized with KX revealed a reduction in the spiking preference in relation to the phase of the oscillation (Figure 5A, *r* = 0.56, *p* < 0.05). Also the phase-frequency distribution of spikes from olivary neurons in the Cx36^−/−^ mutants was shifted compared to spikes recorded from olivary neurons in wild type (WT) mice under KX anaesthetic conditions (Figures 2A and 5A; WT: 98 ± 5°, Cx36^−/−^: 123 ± 8°; *p* < 0.05). On average, the olivary spikes of Cx36^−/−^ mutants expressed 2.3 ± 0.1 wavelets (*n* = 83 spikes) under KX anaesthesia. This value was not significantly different compared to the value obtained from the olivary neurons of WT mice under either KX or MMF anaesthesia (*p* = 0.38 and *p* = 0.30, respectively). In the recordings from mutant mice, we did not detect a significant modulation of the number of wavelets in relation to the phase of the oscillation (Figure 5B, all *p* > 0.05). Thus, the phase of the SSTO did also not modulate the number of olivary wavelets in our *in vivo* Cx36^−/−^ preparations exhibiting a weakened relationship between the oscillation phase and the spiking preference. However, different from all our previous results, the amplitude of the oscillation and number of olivary spike wavelets were not significantly correlated in Cx36^−/−^ mutants (Figure 5C, *r*_CX36_ = −0.20, *p* > 0.05). Therefore, we conclude that under *in vivo* conditions, gap junction coupling via Cx36 may be involved in the modulatory effect of the amplitude of oscillation.

**Figure 6:**
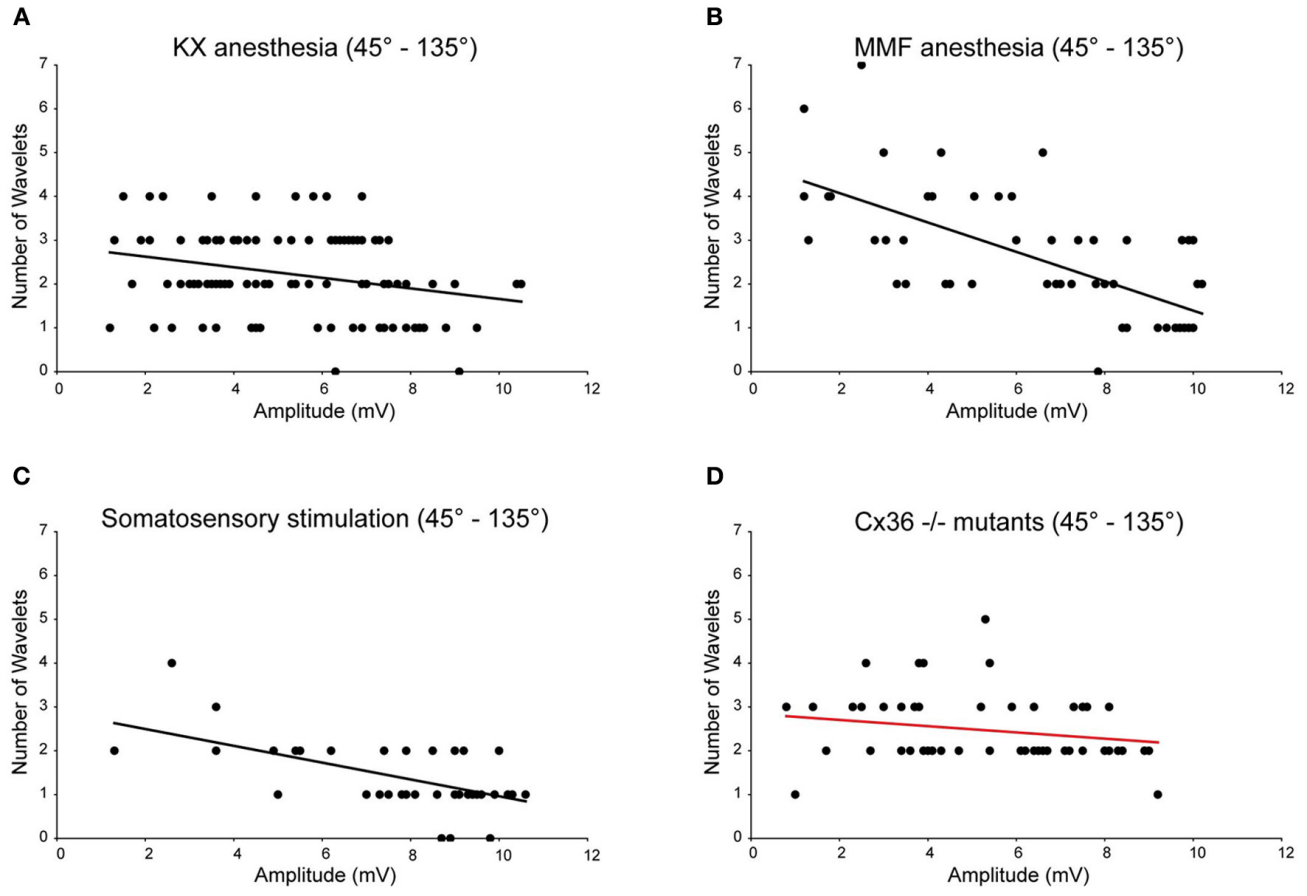
**Burst size of olivary wavelets during the peak phase (45–135°) of the SSTOs.** Relationships between number of wavelets and amplitude of SSTO were plotted of spikes that occur during the peak phase (45–135°) of the SSTOs. Least squares linear regression lines and correlation coefficients were computed for all four conditions (KX anesthesia, MMF anesthesia, somatosensory-evoked action potentials and Cx36^−/−^). Amplitude of the oscillation and number of olivary spike wavelets are significantly correlated under KX conditions [**A**; *r*_KX_ = −0.27, *n* = 110 (71%), *p* < 0.01 (*t*-test)] and under MMF conditions [**B**; *r*_MMF_ = −0.67, *n* = 56 (77%), *p* < 0.01 (*t*-test)]. Amplitude of the oscillation and number of olivary spike wavelets are significantly correlated when measured on somatosensory evoked spikes [**C**; *r*_SSS_ = −0.55, *n* = 44 (56%), *p* < 0.01 (*t*-test)] and are not significantly correlated when measured in Cx36^−/−^ mutant mice [**D**; *r*_CX36_ = −0.21, *n* = 53 (64%), *p* > 0.05 (*t*-test)].

In all four conditions (KX anesthesia, MMF anesthesia, somatosensory-evoked action potentials and Cx36^−/−^), the majority of the action potentials occur during the peak of the oscillation (~45–135°). In order to analyze the impact of the oscillation amplitude on the number of wavelets, we selected and pooled the action potentials of the 45–90° and 90–135° phase subsets for further analysis and cross-conditional comparisons. Consequently, we removed any putative confounding factors of other phase-subsets. Also under these new constraints, the amplitude of oscillation and number of wavelets were significantly correlated to each other in three out of four conditions (Figure 6; *r*_KX_ = −0.27, *r*_MMF_ = −0.67, *r*_SSS_ = −0.55, all *p* < 0.01; *r*_CX36_ = −0.21, *p* > 0.05), confirming the amplitude dependency under all circumstances except in animals lacking electrical coupling. Cross-conditional comparisons of the regression lines revealed a significant difference in amplitude dependency between these groups (*p* < 0.01, ANCOVA). Further analyses revealed that the linear regression line made from the MMF data (the slope as well as the intercept) was significantly different from the linear regression lines of the other conditions (all *p* < 0.05, Tukey's HSD test).

### Harmaline

**Figure 7:**
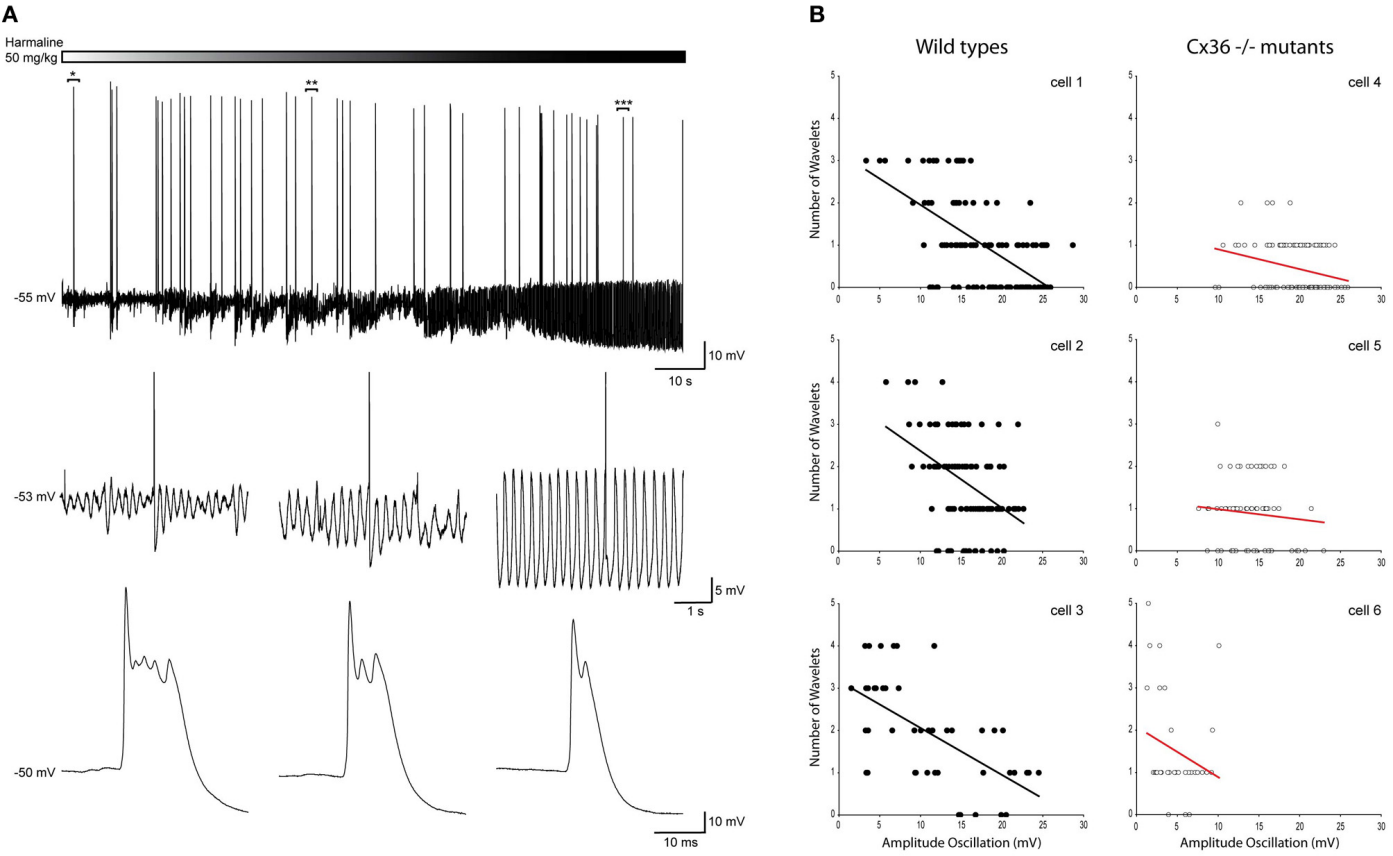
**Harmaline induced alteration of the oscillation amplitude confirms the negative relationship between the amplitude of the SSTOs and the number of wavelets on the ADP of olivary spikes within a single cell. (A)** Top panel: trace of a spontaneous sinusoidal subthreshold oscillating olivary neuron (*in vivo*) during the application of harmaline. Middle panel: three magnifications obtained from the trace above. Magnifications were taken from the marked time windows (^*^, ^**^, and ^***^) and show the effect of harmaline on the subhreshold oscillation. Lower panel: enlargement of the olivary action potential shown in the panel above. The number of the olivary wavelets on top of the afterdepolarization declines, while the amplitude of the oscillation increases. **(B)** Correlation between the amplitude of the SSTOs and the number of wavelets on the ADP of olivary spikes measured in cells from wild type (left panel) and from Cx36^−/−^ mutant mice (right panel). Least squares linear regression lines and correlation coefficients were computed from each cell. Wild types (left panel) cell 1: *r* = −0.59, *n* = 116, and *p* < 0.01 (*t*-test); cell 2: *r* = −0.55, *n* = 143, and *p* < 0.01 (*t*-test); cell 3: *r* = −0.63, *n* = 46, and *p* < 0.01 (*t*-test). Cx36^−/−^ mutants (right panel) cell 4: *r* = −0.04, *n* = 223, and *p* > 0.05 (*t*-test); cell 5: *r* = −0.14, *n* = 74, and *p* > 0.05 (*t*-test); cell 6: *r* = −0.23, *n* = 35, and *p* > 0.05 (*t*-test).

So far, this correlation was obtained by combining the results from many cells. This was necessary because under our *in vivo* conditions, the variability of the oscillation amplitude within a single cell is limited. In order to investigate the causality of this relationship further, we manipulated the amplitude of the oscillations by injecting mice with harmaline (50 mg/kg) during the recordings and correlated the number of wavelets with the manipulated amplitude of the oscillation of a single cell. In all recorded cells (*n* = 6), harmaline increased the amplitude of the oscillation but did not affect the firing rate (Figure 7A). Single cell analysis of data collected from harmaline-injected WT animals (*n* = 3) revealed also significant correlations between the amplitude of the SSTO and the number of wavelets (Figure 7B, all three WTs *p* < 0.01), confirming the inverse relationship between the amplitude of the oscillation and the number of wavelets but now demonstrated within single cells. The three cells that were collected from Cx36^−/−^ mutants did not show any significant correlations (Figure 7B, all three Cx36^−/−^ *p* > 0.05), which also agrees with our results mentioned previously. Thus, manipulating the amplitude of the oscillation from small to large reduced the probability of expressing wavelets (i.e., a smaller burst in olivary axons/climbing fibers), and this causal relationship was not observed in olivary neurons lacking Cx36 gap junctions.

### Low-threshold Ca^2+^ depolarizations affect the generation of olivary wavelets

**Figure 8:**
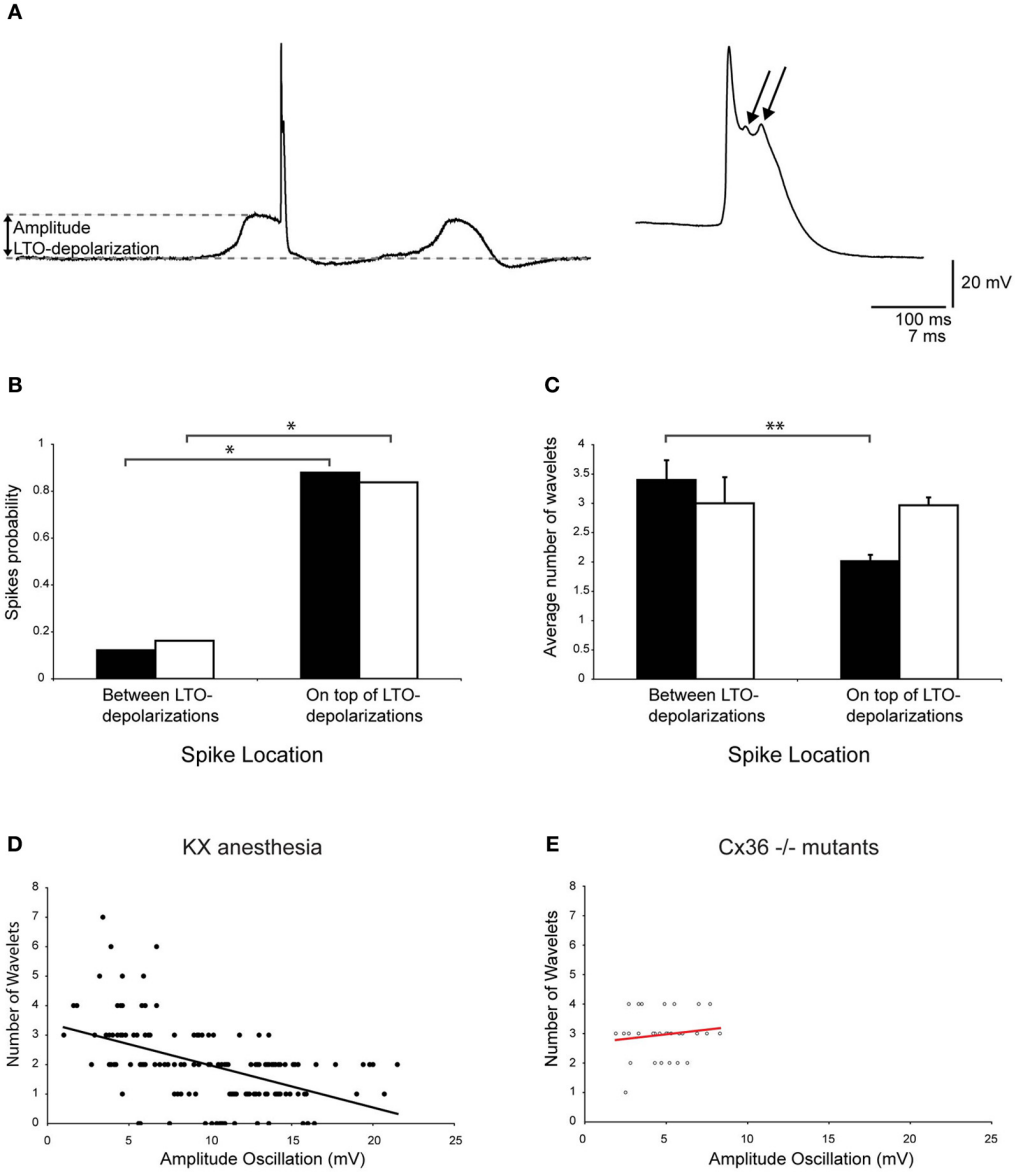
**Modulation of olivary wavelets by low-threshold Ca^2+^ depolarizations. (A)** Left panel: trace of a spontaneous low-threshold Ca^2+^ depolarizations from an *in vivo* olivary neuron. The gray dashed lines indicate the amplitude of the low-threshold Ca^2+^ depolarization. Right panel: enlargement of the olivary action potential shown in the left panel. The arrows indicate the olivary wavelets on top of the afterdepolarization. **(B)** The occurrence of spikes (spike probability) in relation to spike position relative to the low-threshold Ca^2+^ oscillations obtained from recordings measured from either wild type (black bars, *n* = 165) or Cx36^−/−^ mutant mice (open bars, *n* = 37). ^*^p < 0.05 (χ^2^-test). **(C)** Average number of wavelets on the ADP of olivary spikes in relation to spike position relative to the low-threshold Ca^2+^ oscillations obtained from recordings measured from either wild type (black bars, *n* = 165) or Cx36^−/−^ mutant mice (open bars, *n* = 37). ^**^p < 0.01 (*t*-test). **(D**,**E)** Correlation between the amplitude of the LTOs and the number of wavelets on the ADP of olivary spikes measured under KX anaesthesia **(D)** in wild type and Cx36^−/−^ mutant mice **(E)**. Least squares linear regression lines and correlation coefficients were computed from each data set. KX anaesthesia: *r* = −0.51, *n* = 145, and *p* < 0.01 (*t*-test); Cx36^−/−^ mutants: *r* = 0.14, *n* = 31, and *p* > 0.05 (*t*-test).

In addition to neurons that exhibit SSTO, the IO also contains neurons that express rhythmic 1–3 Hz LTOs. Because this type of oscillation was never observed in mice that were anesthetized with MMF, the following analyses were only conducted for olivary neurons recorded from mice that were anesthetized by KX. Spontaneous action potentials in olivary neurons that exhibited LTOs revealed a significant preference for spiking on top of the low-threshold Ca^2+^ depolarizations (Figures 8A,B, *p* < 0.05). Because the waveform of the LTO cannot be fitted correctly to a sine wave, it is impossible to determine the oscillation phase and subsequently the phase-spiking relationship. Therefore, wavelet analysis was performed using a simpler bimodal approach. The number of wavelets was counted on the ADP of spontaneous olivary spikes and grouped for the spikes that were on top of and in between the low-threshold Ca^2+^ depolarizations. On average, these olivary spikes also expressed 2.2 ± 0.1 wavelets (*n* = 165 spikes) under KX anaesthesia, but the number of wavelets was significantly higher in olivary spikes that were not elicited by a low-threshold Ca^2+^ depolarization (Figure 8C, *p* < 0.01).

Olivary neurons that were not interconnected to one another via Cx36 gap junctions showed a preference for spiking on top of the low-threshold Ca^2+^ depolarizations (Figure 8B, *p* < 0.05), similar to olivary neurons that expressed Cx36. However, the olivary neurons recorded from Cx36^−/−^ mutants did not modulate the number of wavelets in a manner dependent on the spike timing in relation to the LTO (Figure 8C, *p* = 0.9).

The bimodal analysis of the relationship between spike timing and the number of olivary wavelets was relatively crude. Therefore, we correlated the number of wavelets with the size of the low-threshold Ca^2+^ depolarization. A significant correlation between the size of the low-threshold Ca^2+^ depolarization and the number of wavelets was found (Figure 8D, *p* < 0.01). These correlations revealed a negative relationship between the size of the low-threshold Ca^2+^ depolarization and the number of wavelets. In the Cx36^−/−^ mutants, no correlation was detected (Figure 8E, both *p* > 0.05). However, the size and number of the low-threshold Ca^2+^ depolarizations were limited in this Cx36^−/−^ mutant study (Figure 8E).

Overall, the size of the olivary axonal burst can be regulated, but it depends on the amplitude of subthreshold oscillation or size of the low-threshold Ca^2+^ depolarization that is expressed by the olivary neuron. Furthermore, gap junction coupling via Cx36 may be involved in this process.

## Discussion

We demonstrated that the phase of the olivary SSTO did not regulate the number of wavelets in olivary bursts under *in vivo* conditions. Instead, we found that the number of wavelets in olivary bursts correlated with the amplitude of the olivary subthreshold oscillation. In addition, this property was not observed in olivary neurons that lacked Cx36 gap junctions. These findings change the current view regarding the nature of the information that is transmitted from the IO to Purkinje cells.

### Number of wavelets: modulation by phase or amplitude?

Our electrophysiological recordings confirm that olivary neurons can generate wavelets under *in vivo* conditions, which was first reported by Crill (1970). The average number of olivary wavelets observed in the present study is similar to previously reported results obtained both *in vivo* and *ex vivo* (Crill, 1970; Mathy et al., 2009). We found that the timing of the spike in relation to the phase of these spontaneous subthreshold oscillations did not determine the number of olivary wavelets in our *in vivo* preparation. It is, though, important to note that in the trough of the oscillation phase bins are present in which no spikes occurred and therefore no wavelets were generated. This scenario is substantially different from a spike that generates no wavelets, because in the first case there is an absence of signal whereas in the latter case the number of wavelets is completely down modulated. This result is inconsistent with the *ex vivo* findings of Mathy et al. (2009). This opposite result might be due to differences in the experimental design. First, Mathy et al. (2009) injected an artificial fixed-amplitude sinusoidal current into the soma to compensate for the lack of spontaneous subthreshold oscillations in their *ex vivo* preparation. Such an induced subthreshold oscillation does not mimic the spontaneous subthreshold oscillation generated by a rhythmic ensemble of dendritic high-threshold and somatic low-threshold Ca^2+^ conductances together with a Ca^2+^-dependent potassium conductance and an H-conductance (Llinás and Yarom, 1986). Second, the IO is isolated in the slice preparation. Removal of the IO from the olivocerebellar loop changes the physiology of olivary cells (Chorev et al., 2007; Khosrovani et al., 2007; Bracha et al., 2009). The observed phase modulation in olivary cells could be due to one or both of these altered physiological conditions. The potential drawback of the *in vivo* preparation is the use and influence of anaesthetics. To address this issue, we used two different kinds of anaesthetics: KX and MMF. Neither condition revealed a phase modulation of the number of wavelets across the phase bins in which spikes occurred. Despite their different influences on a variety of membrane properties, the effects on the phase modulation of the number of wavelets were unambiguous. This result indicates that the type of anaesthetic used does not affect this process. Furthermore, similar results were obtained by analyzing somatosensory-evoked action potentials, suggesting no additional effects by cellular responses related to input processing.

In the present study, however, we provide direct evidence that the number of olivary wavelets is related to the oscillation amplitude. Interestingly, oscillations with small amplitudes showed larger numbers of olivary wavelets compared to oscillations with large amplitudes. To our knowledge, this is the first study to report that the amplitude of the subthreshold oscillation can modulate wavelet number in olivary cells. Both anaesthetics (KX and MMF) revealed an amplitude dependency of the number of wavelets, but the dependency on amplitude was also significantly stronger under MMF than under KX conditions, indicating a possible role for NMDA and/or GABA receptors in this process. The existence of this relationship was confirmed by using Harmaline to manipulate the amplitude of the subthreshold oscillations. The drug is known to cause an 8–14 Hz tremor in mice (Wang and Fowler, 2001) and it is assumed to act by modulating the rhythm-generating ionic currents of IO cells (Llinás and Yarom, 1986; Choi et al., 2010; Park et al., 2010). In our experimental set-up Harmaline caused a prominent increase in the amplitude of the oscillations, without affecting the frequency of the oscillations and the firing frequency. The data collected in these experiments demonstrate the causality of the inverse relationship between oscillations amplitude and number of wavelets, and agrees with the results obtained during physiological oscillations. We also demonstrated that this inverse relationship was not present in olivary neurons that lacked Cx36 gap junction coupling to other olivary neurons. Although gap junction coupling is not necessary for the generation and maintenance of olivary oscillations, it can clearly affect the oscillatory properties of IO cells (Long et al., 2002; De Zeeuw et al., 2003; Leznik and Llinas, 2005). However, it is not yet clear how (mechanistically) the gap junctions are involved in the amplitude-dependent modulation of the wavelets.

### Functional relevance of climbing fiber burst modulation

We have demonstrated that the amplitude, but not the phase, of olivary oscillations modulates the climbing fiber bursts. This mechanism allows olivary axons to convey information about the amplitude of the olivary subthreshold oscillation to Purkinje cells. In the present results, the olivary axonal bursts are smaller when the spike is evoked on a subthreshold oscillation with large amplitude than when it is evoked on a subthreshold oscillation with small amplitude. Studies of Gellman et al. (1985) and Andersson and Armstrong (1987) revealed that the IO can function as an “unexpected” event detector; complex spikes were induced in animals that received an “unexpected” perturbation during their movement. We hypothesize that from the movement command an expected sensory profile is generated. The IO compares this expected sensory profile with the achieved sensory profile that is generated during the movement. An unexpected perturbation induces a mismatch between these two profiles and consequently the IO cells generate a burst of action potentials. We suggest that this expected sensory profile is encoded in the amplitude of the subthreshold oscillations in that a high level of “expectation” is reflected in high amplitude of the oscillation. Or in other words, low-amplitude oscillations make the olivary neurons more likely to respond strongly to signals with a high teaching level. Subsequently, olivary bursts are relayed to Purkinje cells, where they can modify the synaptic transmission between parallel fibers and Purkinje cells (Mathy et al., 2009) and adjust motor performance. According to this theory, the inability of olivary neurons without Cx36 to modulate the axonal bursts should, in Cx36 mutant mice, result in impairment in discriminating between the occurrences of expected and unexpected events during movements. Both locomotion and eye-blink conditioning experiments in Cx36^−/−^ mutant mice revealed that these mice were indeed not able to correctly convert the associated conditional tone into an expected sensory event (Van Der Giessen et al., 2008), which is in line with our hypothesis.

Thus, the amplitudes of olivary subthreshold oscillations might provide a mechanism to grade the expectancy of an event. This information is conveyed by olivary axons to Purkinje cells and is encoded by the size of the burst. Because amplitude modulation of the olivary oscillations in Cx36^−/−^ neurons cannot regulate the burst size, we conclude that the Cx36 gap junction coupling of olive cells is necessary for this mechanism.

## Author contributions

All experiments were performed at the Erasmus MC. Paolo Bazzigaluppi, Jornt R. De Gruijl, and Marcel T. G. de Jeu designed the experiments, Ruben S. van der Giessen, Sara Khosrovani, and Paolo Bazzigaluppi performed the experiments. Paolo Bazzigaluppi, Jornt R. De Gruijl, and Marcel T. G. de Jeu analysed the experiments, Paolo Bazzigaluppi, Jornt R. De Gruijl, Chris I. De Zeeuw, and Marcel T. G. de Jeu wrote the manuscript. All authors approved the final version.

### Conflict of interest statement

The authors declare that the research was conducted in the absence of any commercial or financial relationships that could be construed as a potential conflict of interest.

## Abbreviations

IO: inferior olive
Cx36: connexin 36
C57BL/6 mice: C57 black 6 mice
WT: wild type
KX: ketamine and xylazine
MMF: medetomidine, midazolam, and fentanyl
SSTO: sinusoidal subthreshold oscillation
LTO: low-threshold calcium (Ca^2+^) oscillation
ADP: afterdepolarization
SEM: standard error of the mean.

## References

[B1] Andersson G. Armstrong D. M. (1987). Complex spikes in Purkinje cells in the lateral vermis (b zone) of the cat cerebellum during locomotion. J. Physiol. 385, 107–134 3656160 10.1113/jphysiol.1987.sp016487 PMC1192340

[B2] Bracha V. Zbarska S. Parker K. Carrel A. Zenitsky G. Bloedel J. R. (2009). The cerebellum and eye-blink conditioning: learning versus network performance hypotheses. Neuroscience 162, 787–796 10.1016/j.neuroscience.2008.12.042 19162131 PMC2822538

[B3] Choi S. Yu E. Kim D. Urbano F. J. Makarenko V. Shin H. S. (2010). Subthreshold membrane potential oscillations in inferior olive neurons are dynamically regulated by P/Q- and T-type calcium channels: a study in mutant mice. J. Physiol. 588, 3031–3043 10.1113/jphysiol.2009.184705 20547676 PMC2956943

[B4] Chorev E. Yarom Y. Lampl I. (2007). Rhythmic episodes of subthreshold membrane potential oscillations in the rat inferior olive nuclei *in vivo*. J. Neurosci. 27, 5043–5052 10.1523/JNEUROSCI.5187-06.2007 17494690 PMC6672369

[B5] Crill W. E. (1970). Unitary multiple-spiked responses in cat inferior olive nucleus. J. Neurophysiol. 33, 199–209 4313283 10.1152/jn.1970.33.2.199

[B6] Crill W. E. Kennedy T. T. (1967). Inferior olive of the cat: intracellular recording. Science 157, 716–718 10.1126/science.157.3789.716 6028050

[B7] De Zeeuw C. I. Chorev E. Devor A. Manor Y. Van Der Giessen R. S. De Jeu M. T. (2003). Deformation of network connectivity in the inferior olive of connexin 36-deficient mice is compensated by morphological and electrophysiological changes at the single neuron level. J. Neurosci. 23, 4700–4711 12805309 10.1523/JNEUROSCI.23-11-04700.2003 PMC6740782

[B8] De Zeeuw C. I. Simpson J. I. Hoogenraad C. C. Galjart N. Koekkoek S. K. Ruigrok T. J. (1998). Microcircuitry and function of the inferior olive. Trends Neurosci. 21, 391–400 10.1016/S0166-2236(98)01310-1 9735947

[B9] Desclin J. C. (1974). Histological evidence supporting the inferior olive as the major source of cerebellar climbing fibers in the rat. Brain Res. 77, 365–384 10.1016/0006-8993(74)90628-3 4136782

[B10] Eccles J. C. Llinas R. Sasaki K. (1966). The excitatory synaptic action of climbing fibres on the purinje cells of the cerebellum. J. Physiol. 182, 268–296 5944665 10.1113/jphysiol.1966.sp007824 PMC1357472

[B11] Gellman R. Gibson A. R. Houk J. C. (1985). Inferior olivary neurons in the awake cat: detection of contact and passive body displacement. J. Neurophysiol. 54, 40–60 4031981 10.1152/jn.1985.54.1.40

[B12] Ito M. Simpson J. I. (1971). Discharges in Purkinje cell axons during climbing fiber activation. Brain Res. 31, 215–219 10.1016/0006-8993(71)90648-2 4328277

[B13] Khosrovani S. Van Der Giessen R. S. De Zeeuw C. I. De Jeu M. T. (2007). *In vivo* mouse inferior olive neurons exhibit heterogeneous subthreshold oscillations and spiking patterns. Proc. Natl. Acad. Sci. U.S.A. 104, 15911–15916 10.1073/pnas.0702727104 17895389 PMC2000380

[B14] Lang E. J. Sugihara I. Llinas R. (1996). GABAergic modulation of complex spike activity by the cerebellar nucleoolivary pathway in rat. J. Neurophysiol. 76, 225–275 8836223 10.1152/jn.1996.76.1.255

[B15] Leznik E. Llinas R. (2005). Role of gap junctions in synchronized neuronal oscillations in the inferior olive. J. Neurophysiol. 94, 2447–2456 10.1152/jn.00353.2005 15928056

[B16] Llinás R. Baker R. Sotelo C. (1974). Electrotonic coupling between neurons in cat inferior olive. J. Neurophysiol. 37, 560–571 4827022 10.1152/jn.1974.37.3.560

[B17] Llinás R. Yarom Y. (1981). Properties and distribution of ionic conductances generating electroresponsiveness of mammalian inferior olivary neurones *in vitro*. J. Physiol. 315, 569–584 7310722 10.1113/jphysiol.1981.sp013764 PMC1249399

[B18] Llinás R. Yarom Y. (1986). Oscillatory properties of guinea-pig inferior olivary neurones and their pharmacological modulation: an *in vitro* study. J. Physiol. 376, 163–182 3795074 10.1113/jphysiol.1986.sp016147 PMC1182792

[B19] Long M. A. Deans M. R. Paul D. L. Connors B. W. (2002). Rhythmicity without synchrony in the electrically uncoupled inferior olive. J. Neurosci. 22, 10898–10905 12486184 10.1523/JNEUROSCI.22-24-10898.2002 PMC2834587

[B20] Maruta J. Hensbroek R. A. Simpson J. I. (2007). Intraburst and interburst signaling by climbing fibers. J. Neurosci. 27, 11263–11270 10.1523/JNEUROSCI.2559-07.2007 17942720 PMC6673016

[B21] Mathy A. Ho S. S. Davie J. T. Duguid I. C. Clark B. A. Hausser M. (2009). Encoding of oscillations by axonal bursts in inferior olive neurons. Neuron 62, 388–399 10.1016/j.neuron.2009.03.023 19447094 PMC2777250

[B22] Mauk M. D. Medina J. F. Nores W. L. Ohyama T. (2000). Cerebellar function: coordination, learning or timing? Curr. Biol. 10, R522–R525 10898992 10.1016/s0960-9822(00)00584-4

[B23] Park Y. G. Park H. Y. Lee C. J. Choi S. Jo S. Choi H. (2010). Ca(V)3.1 is a tremor rhythm pacemaker in the inferior olive. Proc. Natl. Acad. Sci. U.S.A. 107, 10731–10736 10.1073/pnas.1002995107 20498062 PMC2890811

[B24] Simpson J. I. Wylie D. R. De Zeeuw C. I. (1996). On climbing fiber signals and their consequence(s). Behav. Brain Sci. 19, 380–394

[B25] Sotelo C. Llinas R. Baker R. (1974). Structural study of inferior olivary nucleus of the cat: morphological correlates of electrotonic coupling. J. Neurophysiol. 37, 541–559 4827021 10.1152/jn.1974.37.3.541

[B26] Szentágothai J. Rajkovits K. (1959). The origin of the climbing fibers of the cerebellum. Z. Anat. Entw. Gesch. 121, 130–141

[B27] Thach W. T. Jr. (1967). Somatosensory receptive fields of single units in cat cerebellar cortex. J. Neurophysiol. 30, 675–696 6035687 10.1152/jn.1967.30.4.675

[B28] Van Der Giessen R. S. Koekkoek S. K. Van Dorp S. De Gruijl J. R. Cupido A. Khosrovani S. (2008). Role of olivary electrical coupling in cerebellar motor learning. Neuron 58, 599–612 10.1016/j.neuron.2008.03.016 18498740

[B29] Wang G. Fowler S. C. (2001). Concurrent quantification of tremor and depression of locomotor activity induced in rats by harmaline and physostigmine. Psychopharmacology (Berl.) 158, 273–280 10.1007/s002130100882 11713617

